# Components of case management in caring for patients with dementia: a mixed-methods study

**DOI:** 10.1186/s12912-022-00935-w

**Published:** 2022-06-23

**Authors:** Desirée Jerez-Barranco, Laura Gutiérrez-Rodríguez, Juan Carlos Morilla-Herrera, Magdalena Cuevas Fernandez-Gallego, Remedios Rojano-Perez, María Dolores Camuñez-Gomez, José Luis Sanchez-Del Campo, Silvia García-Mayor

**Affiliations:** 1 Faculty of Health Sciences, Department of Nursing (Spain), University of Málaga, C/Arquitecto Francisco Peñalosa, 3, Campus Universitario de Teatinos, 29071 Málaga, Spain; 2 Andalusian Health Service, District Costa del Sol, Málaga, Spain; 3 Institute of Biomedical Research in Malaga (IBIMA), Málaga, Spain; 4 Andalusian Health Service, District Málaga-Guadalhorce, Málaga, Spain

**Keywords:** Dementia, Caregivers, Nurses, Case management

## Abstract

**Background:**

Case management has shown improvements in some health outcomes for dementia patients and their families. However, despite its benefits the components of case management in order to provide effective patient and family care remain unknown at present. Thus, the aim of this study is to identify the specific components of case management in caring for patients with dementia and to determine the necessary intensity of its deployment to enhance outcomes for these patients and their caregivers.

**Methods:**

Mixed-methods study with a qualitative phase to characterise forms of service provision, according to the case management components involved, followed by a quantitative phase to analyse the correlations between different patterns of service provision, adverse events in patients and caregiver overload. This study will be based on the variables described in the RANGE.COM register.

**Discussion:**

This research is expected to achieve a reproducible, evaluable set of interventions that can be modelled to optimise case management effectiveness for patients with dementia**.** Interactions between patients with dementia, their family caregivers and case management healthcare services, the components of these interactions and their association with the conditions of the individuals concerned are issues of great interest in the field of case management, which is constantly evolving.

## Background

In recent decades, population aging has increased in line with life expectancy, improvements in the quality and conditions of life and the falling birth rate. The proportion of the global elderly is projected to increase from 12 to 22%, creating a population of two billion men and women by 2050 [1]. In Spain, the National Institute of Statistics (INE) forecasts that the number of persons aged over 80 years will have increased by 260% by 2049 [2].

In Andalusia (southern Spain), the life expectancy of women was 84.9 years in 2019, while that of men was 79.6 years [3]. However, advanced age and chronic disease can provoke frailty and dependence [1] with the consequent risk of hospitalisation, institutionalisation and death [4–6]. The appearance of dementia is usually associated with aging. Its prolonged duration increases morbidity and mortality, reduces the quality of life of patients and their families, provokes many limitations and often creates dependency.

Pathologies causing significant cognitive impairment, such as dementia, have been identified as predictive factors of institutionalisation [7]. Thus, 40% of those admitted to nursing homes suffer from dementia. In Andalusia, an estimated 60,000 people suffer from Alzheimer's disease. Of these, 90% live with their families and 40% are totally dependent [8].

The morbidity and mortality attributed to dementia is increasing worldwide [3, 9–13], and this tendency is expected to increase exponentially [3, 14]. Moreover, the number of hospital admissions for persons with dementia may be ten times greater than that for persons of the same age without dementia [15].

In 2015, the total global cost of dementia was around $818 billion [16], with direct socio-health economic costs of $487 billion, equivalent to 0.65% of the global gross domestic product (GDP) [17]. In 2016, the average annual cost of treatment in Spain for a patient with dementia was €24,184 [18–20].

In the EU, 44.2% of persons aged over 65 years presented some form of disability requiring assistance [21]. In Andalusia, around 66% of dependents were aged over 65 years, these rates being highest in the provinces of Malaga and Seville [22].

This escalation of dependency is associated with an increased need for care, and it is the family environment, not the health system, that is the main provider. In Spain, the White Paper on Dependency [23] defines informal care as “the provision of health care to dependent persons by relatives, friends or others in the immediate social circle, who do not receive financial compensation for the help given”. Caregivers often dedicate a large part of their available time to the direct and indirect tasks involved, with no formal timetable, schedule or direct economic return for doing so, and very often lack the skills required to perform this function, despite the personal costs incurred. In total, the estimated costs for a person with dementia exceed €60,000 per year [24]. Nevertheless, informal care reduces public spending on health care by almost four times [25], and is equivalent to 1.73–4.90% of GDP [26].

Primary caregivers may present fatigue, insomnia, mood swings, anxiety, sadness, irritability or deteriorated social relationships, among other signs and symptoms related to the “overburdened caregiver” syndrome [27, 28]. The patient’s degree of functional dependence and the behavioural alterations experienced are key factors in precipitating this overload, which can lead to the caregiver renouncing this responsibility, and to the patient’s institutionalisation [28, 29].

Health systems, though well prepared to respond to acute situations, are being overwhelmed by chronic health problems [30]. The current failure to overcome this challenge is provoking avoidable hospitalisations, breakdowns in the continuity of care and adverse outcomes for patients [18]. In consequence, the clinical care of acute patients should be complemented with a model that also addresses the issues raised by the chronically sick, requiring comprehensive, continuous and diversified attention [31]. To achieve this, self-care, case management and primary care should be promoted and reinforced as the crucial elements of chronic care [19].

According to the Case Management Society of America, case management is “a collaborative process of assessment, planning, facilitation, care coordination, evaluation, and advocacy for options and services to meet an individual’s and family’s comprehensive health needs through communication and available resources to promote quality cost-effective outcomes” [32]. It may improve home care and help increase service efficiency and effectiveness by boosting patients’ functional recovery, fostering access to professionals and resources, increasing patient and caregiver satisfaction, streamlining therapeutic regimens, reducing caregiver overload and optimising the use of services [33].

Case management may also improve outcomes at certain time points in patients with dementia: for example, it reduces the risk of institutionalisation in the first six and eighteen months of dementia, reduces agitation at eighteen months, reduces the number of days of hospital admissions in the first six and twelve months and reduces the burden on the caregiver in the first six months [34].

Despite these acknowledged benefits, there remains some uncertainty about the specific components of case management and the intensity with which they should be deployed to provide an effective response to dementia. Among other causative factors, this indefinition may be due to the complexity of the interventions performed. A complex intervention, whether therapeutic or preventive, comprises a large number of individual interacting elements, all of which are related to the patient, the organisation (for example, the duration and frequency of interventions) and/or the health care itself (such as the professionals involved and the healthcare context) [35]. However, while these elements may all be essential to proper functioning, it is difficult to isolate the “key ingredients” determining the effectiveness of the intervention. Indeed, the greater the difficulty in defining these key ingredients and how they are interrelated, the greater the probability of the intervention being complex and thus more difficult to reproduce for other contexts, patients or organisations [36].

To address these issues, Campbell et al. [37] developed a conceptual framework to establish guidelines for the correct development of randomised controlled studies aimed at defining and evaluating complex interventions.

Various studies have reported the positive effects of case management in the treatment of dementia [38–50], reduce the fatigue anxiety of caregivers [49], delay institutionalisation [46] and foster the uptake of community services [42].

Regarding the specific components of case management for patients with dementia, Backhouse et al. [51] identified five elements that are determinant to the effectiveness of an intervention: the characteristics of the case management nurses (CMN) involved, communication (among professionals and with their clients), the type of intervention, the resources available and the support network established (again, both for the professionals and for their clients). In related work, Verkade et al. [52] described the following essential components of case management in patients with dementia: information, support and advice, coordination of the care provided and, to a lesser extent, practical help. The main conclusion drawn by these authors was that case management for patients with dementia should assist both the patient and the underlying system of support.

The Andalusian Public Health System (SSPA) has been implementing case management since 2002, a service that is provided by almost 400 professionals, throughout the region. It is one of the pioneering services in Spain and its effectiveness has led many other areas to adopt this reference model [33]. The Andalusian system was originally designed to serve the homebound population, often subject to complex chronic disease. Nevertheless, the real capacity of the Andalusian model to respond to the needs of this population has yet to be clarified.

For these situations, a commonly-adopted approach in health service research is to generate registers of diseases or specific clinical situations. These have a long tradition in cancer research [53] and in many other areas, such as stroke [54], mental health [55] and cardiovascular disease, such as the ARIAM registry [56]. This type of record-based research has great potential for understanding health processes from a longitudinal perspective and for obtaining data under normal practice conditions.

In view of these considerations, our research group created the RANGE.COM multi-centre case management register in Andalusia in 2012 [57]. This register currently includes 1,065 patients, detailing the profile and immediate social context of every patient with multimorbidity who receives this service. At present, RANGE.COM covers part of the internationally standardised outcome criteria for elderly persons with frailty [58], together with some variables related to social and family determinants.

Evidence-based guidelines and recommendations have been published for most of the health problems underlying dementias and appropriate quality measures. However, the transfer into practice of this evidence has not been systematically investigated and there is uncertainty about the structures and processes that would support its implementation and, ultimately, enhance nursing outcomes for persons with dementia [59]. Furthermore, the studies that have been conducted to date present large variations in terms of the case management models and outcome criteria evaluated. Consequently, the results of effectiveness studies remain far from conclusive.

The present study, therefore, addresses the need to analyse the interactions of patients, families and the health services, to describe the components of these interactions and to determine their association with the status of the patient and family. In parallel, we intend to establish a synergy with the information obtained from the RANGE.COM register [57], opening up new options in response to the major research question guiding this study.

## Aims

### General aims

1. To identify the components of case management in persons with dementia and in their primary caregivers, in order to characterise appropriate forms of service delivery.
2. To analyse the correlations between different patterns of service provision, on the one hand, and the presence of adverse events in patients and of burden on family caregivers, on the other.

### Specific aims

1. To describe the sociodemographic and clinical characteristics (such as functionality and comorbidities) of patients with dementia and the characteristics of their caregivers (dedication to care, perceived burden) who receive case management in the home environment, within the portfolio of services provided by the Andalusian Public Health System.
2. To identify common components and interventions used in practice by CMN.
3. To describe the correlation between components / interventions deployed by CMN and the presence of adverse events in patients (mortality, falls, hospital admission, pressure ulcers and institutionalisation).
4. To analyse the correlation between the components / interventions deployed by CMN and the burdens perceived by family caregivers of persons with dementia.

## Methods

These study goals will be addressed by means of a sequential exploratory study of mixed methods in which a first qualitative phase will try to explore the practice patterns implemented by the CMN which it will guide a second quantitative phase. In the first phase it will be created a conceptualized analysis framework with which to study the data obtained. During quantitative phase, the case management results of the range registry will be investigated in relation to the content and distribution of the service components. Hence, the constructivist approach of qualitative methods, thus providing new perspectives and facilitating a better understanding of the characteristics, processes and results of case management for persons with dementia [60].

### Setting

The study will be carried out in Málaga (Spain), where the primary care reference is the Málaga Health District. This city has 26 health centres, with 289 nurses, 319 family doctors and 23 CMN. Of the latter, 91.6% are accredited by the Andalusian Quality Agency and all have completed case management training.

Recruitment for the study will take place at all 26 health centres.

### Participants

Patients recently diagnosed with dementia and their caregivers, referred for case management and willing to participate in the study.

CMN: Eight persons will be selected, in two groups of four, classified by years of experience.

#### Inclusion criterion for patients

Diagnosis of dementia for patients who are new users of the case management services portfolio in the Málaga Health District, not in a terminal situation and accompanied by a family caregiver.

#### Exclusion criterion for patients

Refusal of consent, either directly or through the caregiver.

#### Inclusion criterion for caregivers

Primary caregiver for the patient in question.

#### Exclusion criterion for caregivers

Incapacity to perform this function due to serious mental disorder, such as depression, bipolar affective disorder, schizophrenia, developmental disorders or dementia.

### Data collection

#### Phase 1. Qualitative study

Qualitative, case study design, using participant observation combined with in-depth interviews. The results obtained from Phase 1 will identify the components of case management applied to patients with dementia and their caregivers, from observation of this process in the natural clinical context. This methodological approach will enable us to consider a specific context, with a well- defined organisation and social setting, i.e. the provision of home care for persons with dementia, and will facilitate the involvement of a limited number of participants through episodic observations. Moreover, this approach has been adopted in a previous study of case management components [61]. With this method, we can address specific phenomena to better understand the "hows" and "whys", without manipulating the participants’ normal way of life. Furthermore, relevant contextual factors may be revealed, despite the possible blurring of boundaries between the context and the phenomenon to be studied [62].

##### Qualitative sampling

In this study, the unit of analysis will be the CMN, selected by intentional qualitative sampling to achieve four groups with the following segmentation criteria: relevant experience of more or less than ten years, and the socioeconomic level of the health area considered (low or medium–low vs. medium–high or high). This approach allows us to take a multiple-case approach to identify intra- and inter-case analogies and similarities [62]. Eight health centres will be selected, according to their ranking in the average municipal gross income per 1,000 inhabitants (data provided by the Spanish Finance Ministry [63].

This process will result in the selection of three health centres in the upper quartile of average income, three in the central quartile and three in the lower quartile. In total, eight CMN will be selected for participant observation and in-depth interviews. All patients who meet the criteria for dementia and who receive or request case management services from these nurses will be included in the analysis.

##### Study design

To avoid the usual over-expansion of case studies, we will apply the recommendations of Yin et al. [64]. A temporal and spatial framework will be established for the observation conducted at each health centre, delimited by the activity (patients with dementia who receive or request case management) and the context (home care services). Accordingly, a contact interview will be scheduled between the researcher and the CMN, during a normal working day.

Patients with dementia will be identified by their physician, family nurse or social worker, and referred for coordination by the CMN. Subsequently, the observations will be made on a date agreed with the nurse.

The observation location will usually be the patient's home, although for those in initial GDS-FAST stages, the contact might be made at the health centre. Participant observation will take the form of interaction between researcher and informants, in the latter’s home ground, thus enabling data to be collected in a systematic and non-intrusive way [65]. The presence of the researcher is of decisive importance, but must not alter the normal functioning of the scene observed. The only modification to the scene will be this presence, and the researcher’s role will be strictly non-determining in the social action [66]. To ensure these requirements are met, the observer accompanying the CMN will be a nurse with primary healthcare experience. Therefore, the duration of the familiarisation phase will be minimal.

The participant observation may require several CMN visits until the case episode is considered closed. Resources created for this study include a field journal and a set of observation units, based on a prior review of the literature and on consultation with expert researchers in the field (case management professionals with at least five years’ practice and research experience). Additional units may also be added, if necessary, in the course of the observations. During the observations, formal semi-structured interviews will also be carried out between the CMN and the patients and caregivers. Informal interviews may also be held in the natural environment of the observation. Another potential element is the analysis of documents or records of interest (for example, notes taken by the CMN, exchanges of written information between the parties, protocols, guides and aids to decision making).

All observations and interviews will be recorded with a digital voice recorder, to be used exclusively for this study, with prior written authorisation from the patient, if capable, or otherwise from his/her representative. In addition, authorisation will be obtained from all persons being recorded while accompanying the study subject. Every observation will later be transcribed, as far as possible reflecting the whole tone and content of the recording. Field notes will be taken on aspects related to body and gestural language, the researchers’ reflections, descriptions of persons and contexts and other items considered of interest by the researchers in situ.

The transcription will be performed by members of the research team and stored using ATLAS.Ti 22 qualitative analysis software.

##### Qualitative analysis

The transcription documents, as well as the field notes will be carefully read to identify emerging themes and will form the basis for a deductive coding (code by list), incorporating the components detailed by Verkade et al. [52]. Additionally (as in-vivo coding), further components from outside this list may be added. The encoded fragments will then be refined, modified or even reconfigured, for subsequent categorisation. All non-encoded material will also be reviewed, and if it contains important discourse elements it will be encoded freely.

These actions will generate a list of codes, to be grouped into categories, subcategories and possible topics of analysis.

A second member of the research team will triangulate the encoded information and independently review the encoding performed. Any discrepancies will be resolved by consensus among the researchers.

The results obtained will then be converted into a catalogue of components that can be used to map each aspect of the case management applied.

The credibility and validity of the data will be ensured by applying the reliability criteria proposed by Lincoln and Guba [67]: credibility, transferrability, consistency and neutrality (confirmability).

#### Phase 2. Quantitative study

This phase will consist of an analytical cross-sectional observational study, addressing Study Aims 1, 3 and 4.

##### Study subjects: population and sample

The study sample will consist of 157 CMN and 186 patients with dementia included in the Andalusian Register of patients undergoing follow-up by case management (RANGE.COM) [57] plus those who are currently in follow-up or who enter as new cases. Subsequently, post hoc statistical power estimations will be carried out. This register currently describes 1,065 patients, throughout Andalusia, being followed up by case managers. Any CMN who has no cases with dementia will be excluded from the analysis, although there is little probability of this occurring, due to the high frequency with which these patients occupy the provision of case management attention.

##### Data compilation

First, we will analyse the variables characterising the patients with dementia who are recorded in the RANGE.COM register at the time of the study, in terms of sociodemographics (age, sex, education, cohabitation) and clinical status (functionality, cognitive impairment, comorbidities). We will also address the variables corresponding to the caregivers (age, sex, education, daily dedication to care and burden).

Subsequently, using the case management components of dementias identified in the qualitative phase, a data collection form will be developed addressing the individual components, so that an email invitation may be sent to each of the RANGE.COM registered nurses (listed in this information registry), asking them to select the components that best describe their daily professional practice in patients with dementia. Each component will be self-assessed in order of the frequency with which it is applied in daily practice, scored on a Likert scale ranging from 1 to 5 (1 = I never perform this intervention for any patient or family caregiver; 5 = I perform this intervention regularly for all patients and/or family caregivers).

In addition, the nurses will be asked to provide sociodemographic data (age, sex) and details for professional characterisation (years of total experience, years of experience in case management, academic level achieved and self-perceived level of competence, using the Advanced Practice Nurses Competency Assesment Instrument [APNCAI] for the evaluation of competencies in advanced practice nurses validated by our research group) [68].

These components will then be analysed to generate groupings from which patterns of differential practices can be identified.

##### Quantitative analysis

Descriptive statistics will be obtained, with measures of central tendency, dispersion and the distribution of percentages for the variables characterising the participant nurses and patients. The normality of the distributions will be checked using the Kolmogorov–Smirnov test. Bivariate analyses will be carried out according to this normality or otherwise and the nature of the variables compared, using Pearson’s or Spearman's correlation, the chi-square test, Student's t test or Manuscript-Whitney's U test and ANOVA (after verification of homoscedasticity with the Levene test), applying the Brown-Forsythe and Games-Howell test in cases of heteroscedasticity, and the Kruskall-Wallis test otherwise.

A k-means cluster analysis will be carried out to identify groupings of CMN profiles according to the distances observed between the centroids of the variables generated by the components of the qualitative phase. This analysis will take an exploratory approach, seeking, a priori, to identify 3–4 clusters from which the one deemed most plausible will then be selected. An ANOVA will be performed on the cluster assigned to each CMN to identify associations between practices and outcomes (numbers of readmissions, acute hospitalisations, situations of caregiver overload, falls and visits to healthcare providers – General practitioner [GP], family nurse, social worker, hospital specialist). A chi-square test will also be performed to determine the relationship between the membership cluster and institutionalisation. Finally, a multivariate analysis will be made of outcomes such as institutionalisation, falls, hospital admissions and caregiver overload, using multiple linear regression with hierarchical methods, and incorporating the CMN as a second level of fitting (incorporated within the corresponding cluster). The first level will incorporate the patient’s age, sex and level of comorbidity, together with variables presenting a relevant association in the prior bivariate analyses, in order to detect possible confounders**.**

## Discussion

The results of this study will provide information as to which case management models and interventions are most effective in achieving results beneficial for the health of persons with dementia and for their caregivers, thus reducing the risks of institutionalisation and caregiver overload. The results obtained from this research are expected to be transferable through consensus documents, enabling us to compare and contrast modes of case management practice and to differentiate the results achieved. Foreseeably, these findings could guide recommendations to combine effective intervention packages, although they will need to be examined against other, solid research findings in order to make final recommendations on clinical practice guidelines for healthcare systems. The research results are expected to be widely applicable and will facilitate improvements to routine clinical practice in the Andalusian Public Health System in the following areas:

- Characterise the case management models used in Andalusia for patients with dementia.
- Determine the most effective case management practices and those most benefiting patients’ health and caregivers’ wellbeing.
- Reduce the risk of institutionalisation of persons with dementia.
- Improve the management of personal and family health.
- Reduce the burden on caregivers and healthcare services.
- Increase patient and caregiver satisfaction.

Studies conducted in Andalusia have shown that case management improves home care for patients and increases service efficiency and effectiveness by enhancing the functional recovery of patients, facilitating access to professionals and resources, improving patient and caregiver satisfaction, contributing to the management of treatment regimens and reducing caregiver overload and the use of services [33]. Moreover, various systematic reviews have reported that case management benefits both patients and caregivers, although further research is needed to determine the optimal extent and characteristics of the programme applied [69, 70]. At the global level, results in this field are uneven, probably due to variability in case management practices [71–76].

It has been estimated that by 2030 some 156,000 people will be affected by dementia in Andalusia [77]. Therefore, the research findings we expect to obtain will be applicable to large numbers of patients and their caregivers, both in this region and elsewhere [1].

### Limitations

In any given study, the value of clinical practice models to study their association with the results achieved, using a retrospective database, may be affected by changes in the professional practices during the period considered or by fluctuations in the characteristics of the professionals who provide the service. However, case management is well established in the Andalusian Public Health System and no major modifications are foreseen in this regard, although the possible influence of the contingencies mentioned will be monitored throughout the study.

## Data Availability

Data sharing is not applicable to this article as no datasets were generated or analysed during the current study.

## Abbreviations

INE: National Institute of Statistics
IECA: Andalusian Institute of Statistics and Cartography
GDP: Gross Domestic Product
SSPA: Andalusian Public Health System
CMN: Case Management Nurse
APNCAI: Advanced Practice Nurses Competency Assessment Instrument
GP: General Practitioner
